# Using evidence-based models of human behaviour to reduce endocrine-disrupting chemicals exposure: recommendations for strengthening interventions

**DOI:** 10.12688/openreseurope.21765.2

**Published:** 2026-07-16

**Authors:** Lisa Selma Moussaoui, Ariane Baye, Annick Bogaerts, Alice Collinge, Roland Devlieger, Xavier Escoté, Carmen Freire, Barbara Kaiser, Dorota Komar, Maryse Ledent, Leonie Miserez, Noemi Boqué, HYPIEND Clinical Core Investigator Group, Silvia Orte, Laura Sistach, Antoni Caimari, Salvador Fernandez, Inés Velasco, Paula Sol Ventura, Marta Ocaña, Mirjana Andjelkovic, Ilse Van Overmeire, Adil Mardinoglu, Ozlem Altay, Hugo Vankelecom, Frauke Christ, Fernando Vela-Soria, Raquel Barea, Michal Ciebiera, Alicja Baska

**Affiliations:** 1 Research group in Health Psychology, University of Geneva Faculty of Psychology and Education, Geneva, Geneva, 1205, Switzerland; 2 Research Unit of EQUALE, University of Liege, Liège, Walloon Region, 4000, Belgium; 3 REALIFE Research Group, Research Unit Woman and Child, Department of Development and Regeneration, KU Leuven, Leuven, Flanders, 3000, Belgium; 4 Department of Obstetrics and Gynaecology, University Hospital Leuven, Leuven, Belgium; 5 Unitat de Nutrició i Salut, Technology Centre of Catalonia Eurecat, Barcelona, Catalonia, 43204, Spain; 6 Nutrition and Metabolic Health Research Group. Institute of Health Pere Virgili (IISPV). Center of Environmental, Food and Toxicological Technology – TecnATox, Rovira i Virgili University Department of Biochemistry and Biotechnology, Reus, Catalonia, Spain; 7 Biomedical Research Network Center of the Pathophysiology of Obesity and Nutrition, Madrid, Community of Madrid, Spain; 8 Department of Legal Medicine, Toxicology and Physical Anthropology, University of Granada, Granada, Andalusia, 18016, Spain; 9 Instituto de Investigacion Biosanitaria de Granada, Granada, Andalusia, 18012, Spain; 10 CIBER de epidemiología y Salud Pública, Madrid, Spain; 11 Centre for Omic Sciences (COS), Joint Unit Universitat Rovira I Virgili-EURECAT, Unique Scientific and Technical Infrastructures (ICTS), Technology Centre of Catalonia Eurecat, Barcelona, Catalonia, 43204, Spain; 12 Risk and health impact assessment, SCIENSANO, Brussels, 1050, Belgium

**Keywords:** Endocrine disruptors; Awareness raising; information deficit model; Health Action Process Approach (HAPA); School Wide Positive Behavioural Interventions and Supports (SWPBIS); Behaviour change techniques (BCTs); Lifestyle interventions

## Abstract

Endocrine-disrupting chemicals (EDCs) represent a threat for the ecosystem and human health. Exposure routes include modifiable exposures, such as choice of food or cosmetics but also daily habits such as airing the room. However, current attempts to reduce modifiable exposure often rely on knowledge provision and awareness-raising campaigns. This is the case even though human behaviour, decision making and habits change have been studied for a long time, and numerous psychological theories demonstrate that the transition from awareness of a health threat to actually change daily behaviour is not straightforward. This paper calls for interventions on EDCs exposure reduction to build on solid understanding of human behaviour and use of behaviour change techniques to achieve significant impact. We illustrate how this can be done by presenting the HYPIEND project, a multicomponent behavioural intervention designed to help pregnant women and prepubertal children (and their parents) to reduce their modifiable exposure to EDCs in these sensitive periods. HYPIEND relies on theoretical models such as the Health Action Process Approach (HAPA) model, motivational interviewing techniques, and broader support through partners (for pregnant women) and the school environment (for children) through a school-wide project built on the School Wide Positive Behavioural Interventions Supports.

**Abbreviations list**
BCTs: Behaviour change techniques
EDCs: Endocrine-disrupting chemicals
HAPA: Health Action Process Approach
PFAS: Per- and polyfuoroalkyl substances
RCT: Randomized Controlled Trial
SWPBIS: School Wide Positive Behavioural Interventions and Supports

## Disclaimer

The views expressed in this article are those of the authors. Publication in Open Research Europe does not imply endorsement of the European Commission.

## Context

The Endocrine Society defines an endocrine disrupting chemical (EDC) as: “an exogenous chemical, or mixture of chemicals, that can interfere with any aspect of hormone action”. ^1^ ^,^ ^2^ Among EDCs, some naturally occurring compounds can be found ^3^; however, most of them are synthetic, human-made chemicals. According to estimates of the European Environment Agency, approximately 70% of around 100,000 chemicals on the EU market have been poorly assessed for their safe use. ^4^ A significant number of them have been found to disrupt the endocrine system. As of June 2025, 227 substances were identified as EDCs at the EU level ( List I), and additional 104 substances are currently under evaluation ( List II). Importantly, the Global Chemical Outlook II states that global trends, such as population dynamics, urbanisation, and economic growth, are rapidly increasing chemical use, particularly in emerging economies. ^5^ Recent studies all around the globe have demonstrated the presence of EDCs in the environment. ^6^ ^–^ ^8^

EDCs can have substantial impact on ecosystem and human health. ^1^ For instance, they can induce a wide array of reproductive and developmental disturbances in aquatic species, ^9^ ^,^ ^10^ as well as in birds and mammals. ^9^ Studies conducted in grassland and freshwater ecosystems have demonstrated that EDCs-induced disturbances can lead to reductions in both biomass and the capacity for ecosystem recovery. ^10^ Regarding human health, EDCs can interfere with endogenous hormone biosynthesis, metabolism, or action, disrupting homeostatic and developmental processes. ^11^ Epidemiological and experimental studies have linked EDCs exposure to impaired reproductive function, immune impairment, neurodevelopmental deficits, metabolic disorders, and hormone-dependent cancers. ^12^

Interventions and communication campaigns are developed to reduce EDCs exposure. However, many of them rely on knowledge provision, with the idea that awareness raising is sufficient to induce behaviour change. For example, in their chapter *Policy implication and community interventions to reduce EDCs exposure*, Montano and Guglielmino propose that “information campaigns could be organized for the general population or population particularly exposed to EDCs. An action of this dimension could change exposure levels by informing and raising awareness of how to avoid the possible and different contact with individual EDCs and defend health from the sneaky snare of EDCs”. ^13^ ^p.221^ Decades of work in behavioural sciences have demonstrated how the process is complex ^14^; and provide insights on how people change their habits. This paper is a call to use this scientific knowledge to build interventions that maximise their chances to ultimately have substantial impact on ecosystem and human health. We begin by discussing routes of exposure to EDCs, especially behavioural ones, before reviewing models of behaviour change. We then present examples of successful and less successful studies and campaigns, analysed through the prism of psychological determinants and behaviour change techniques. The HYPIEND project is then presented for illustrative purpose as an example of an intervention aimed at reducing EDCs exposure based on behavioural sciences. Given the trial is currently in progress, no outcome data have been collected yet and no results are reported in this publication. The main aim of the paper is both conceptual and methodological: emphasising the need to go beyond education to induce behaviour change in relation to EDCs exposure and provide an example of a theory-informed intervention.

## Routes of exposure

Human biomonitoring studies repeatedly report wide presence of EDCs in human bodies across various regions in the world. ^15^ People are exposed to EDCs from multiple sources, including environmental pollution (air, ^16^ ^,^ ^17^ water ^18^ ^–^ ^20^ and soil ^21^), but also through consumer products, including food. ^22^ ^,^ ^23^ Early life is a vulnerable period, as exposure begins as early as the peri-conceptional and prenatal periods (through the placenta), ^24^ and postnatally, through breast milk and food. ^25^ Modifiable exposure is broadly understood as a behavioural or environmental factor that is measurable and modifiable by individuals, communities, or governments. ^26^ For example, bisphenols are added to plastics to improve physical, thermal and mechanical properties of the product. Even if the use of bisphenol A in food contact materials was banned in the EU, substitutes are commonly found in water bottles, food containers, and can leach into their contents. ^27^ ^,^ ^28^ An important dermal exposure to bisphenols is often attributed to thermal paper – receipts; and more recently, clothes made from synthetic textiles. ^29^ Phthalates are plasticizers added to plastics to increase flexibility and resistance to high temperatures and to achieve the same properties, to personal care products (to reduce cracking of nail polish, avoid stiffness in hairsprays or as fragrance retainers). ^30^ Per- and polyfluoroalkyl substances (PFAS) are persistent EDCs used in water and stain-free surfaces like non-stick cookware, water-repellent clothing, stain-resistant fabrics and carpets, fast-food wrapping paper; and for smoothing effect in cosmetics. ^31^ Compounds that affect the endocrine system are frequently present in pesticides, as well as in their residues found in conventionally farmed crops and animal-derived foods. Ultra-processed foods are particularly high in these compounds due to processing, packaging, and formulation. ^3^ Flame retardants also represent a significant source of EDCs exposure, and their use has increased in parallel with the rising production and consumption of synthetic textiles. ^32^

Studies have shown that not everybody has the same level of exposure and suggest it can contribute to explain disparities in health outcomes between racial/ethnic groups or according to the level of income notably. ^33^ ^,^ ^34^ For example, Llanos et al. showed that personal care product use varied between females and males, and by ethnicity. ^35^ Govarts et al. ^15^ reported that exposure differed strongly depending on socioeconomical status and geographical location.

## Reducing exposure through behaviour change

Addressing risk factors associated with EDCs requires multifactorial interventions. ^36^ One important approach is to reduce exposure through behaviour change. Individuals can make choices about the type of bottle they use to drink water, how frequently they ventilate their homes, or whether to use certain cosmetics, such as nail polish or perfume. Of course, people also depend on the types of products offered on the market. ^37^ ^,^ ^38^ Therefore, regulatory and industrial actions are essential to reduce EDCs exposure. However, when several options are available, it remains that individuals must be willing to choose EDCs-free products, and beyond willingness, they need to be able to maintain these changes in their consumer habits in the long term. Those notions of willingness and capacity refer to important determinants of behaviour, which have been studied for a long time in psychology.

## Evidence-based models of behaviour change

Since the 1950s, scientists have studied what predicts behaviour. Early work from behaviourists explains that the future likelihood of a behaviour depends on the consequences (positive or negative) that the person experiences when engaging in that behaviour. ^39^ Theories aiming to explain human behaviour later evolved to include not only external influences, but also internal ones, such as the perceived capability of performing a behaviour, ^40^ ^,^ ^41^ beliefs about the consequences of the behaviour, perceived approval by others and intention. ^41^ ^,^ ^42^ Other theories focused on the perceived health threat as a motivator for adopting health behaviours, ^43^ ^,^ ^44^ suggesting that someone will perform a health protective behaviour if they believe they are vulnerable to a threat, perceive the threat as severe, and that they are capable of adopting a protective behaviour. However, reviews and meta-analyses show that threat-related variables do not predict behaviour well. ^45^ ^–^ ^49^

Models later evolved to include explanations about the process from intention to do something to the actual realisation of the behaviour. ^50^ The importance of both motivational (preceding the decision to act) and volitional (following the decision) phases for behaviour change were demonstrated in a meta-analysis. ^51^

Other models focus on the importance of the context explaining that behaviour is shaped by multiple interconnected levels, ranging from the individual to the policy. ^52^ In the case of children’s behaviour, models have considered specific influences, notably the fact that the decision of the action is often not the responsibility of the child, instead they are often asked/required by the adults to do specific behaviours. ^53^

**Figure 1.**
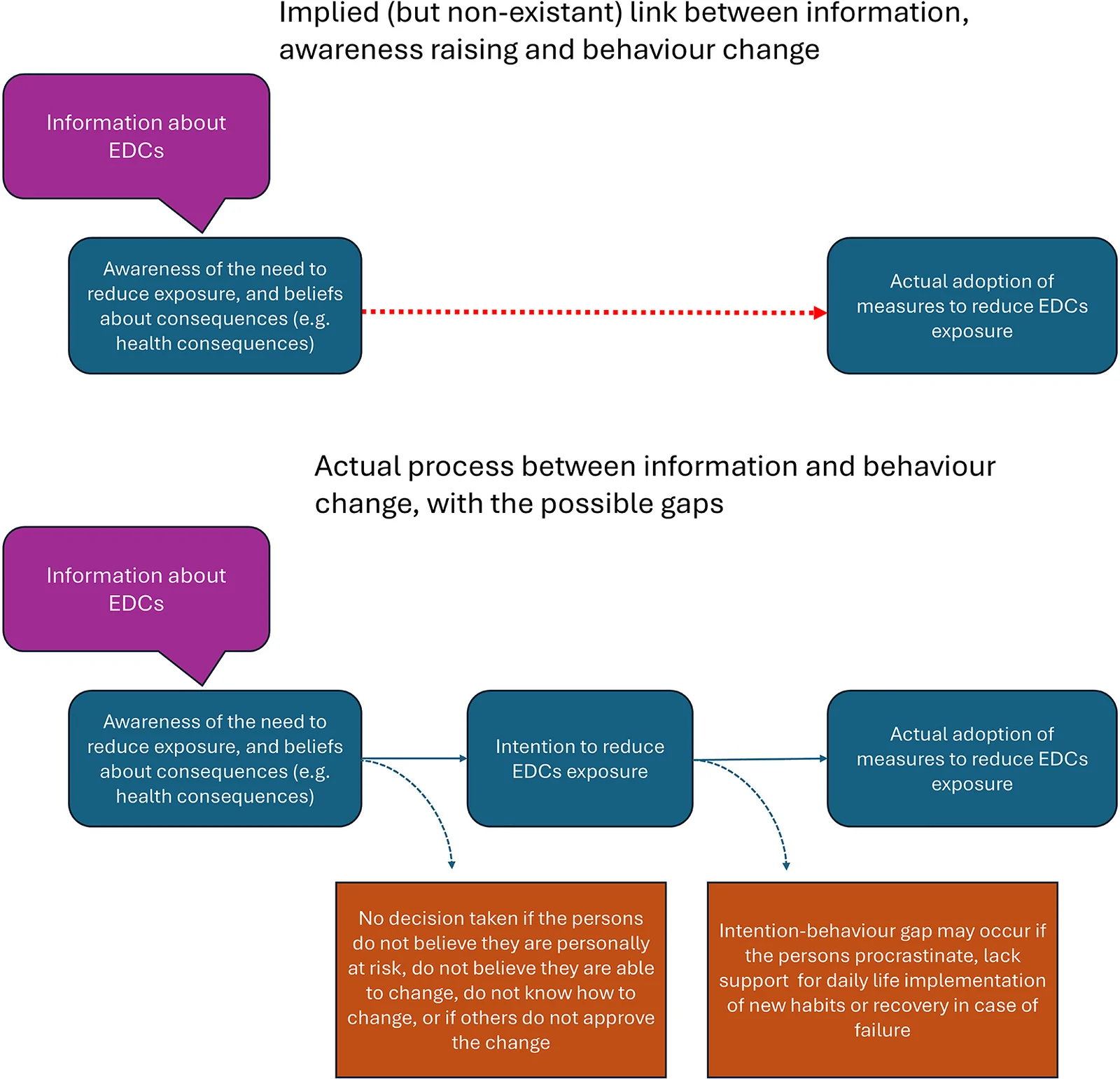
Schematic representation of the process between provision of information and the eventual resulting change in behaviour. The gaps mentioned (in orange) are illustrative and not exhaustive of all possibilities. EDCs = Endocrine Disrupting Chemicals.

This review of various models illustrates that even in the very early models of behaviour, information has never been considered sufficient to change behaviour successfully. Figure 1 illustrates schematically the process from information to behaviour change, incorporating findings from several behaviour change models.

Nevertheless, despite being insufficient, information and knowledge can still be necessary. Greyson and Johnson ^54^ showed how information is included in many of the models of behaviour. According to their analysis, beliefs about the consequences of the behaviour are constructed, at least in part, based on received information, and information can raise awareness of the need for change. In particular, information and knowledge may be needed for issues that people are not yet knowledgeable about. On the topic of EDCs, surveys and qualitative studies among various groups (students, pregnant women, and adults from the general population) indeed show that people lack knowledge about what EDCs are and the associated risks. ^55^ ^–^ ^59^ Correlational studies on the level of knowledge about EDCs and health behaviour showed mixed results: some report no significant association, ^60^ ^,^ ^61^ while another study showed that this association varied depending on the substances. ^62^ Knowledge about parabens was associated with more avoidance of chemicals in personal care and household products, but this was not the case for lead: lead was the most widely recognized substance, but the least avoided. ^62^

## Lifestyle interventions to reduce EDCs exposure

Reviews show that policy interventions (using natural experiment study design to test substances bans effects), as well as interventions that provide low-chemical personal care products, ^63^ plastic-free foodware, organic food, or fresh food to the intervention group compared to canned food for the control group, ^64^ succeed in reducing the levels of chemicals measured among participants. However, controlled consumption studies attribute a passive role to participants and do not reflect real life conditions in which individuals have to make choices. This limitation is underlined in the review by Park *et al*., ^65^ showing that only four studies among the ones reviewed let participants choose themselves alternative goods or food. In addition, among the 12 interventions reviewed, half lasted less than ten days, ^65^ failing to assess temporal dynamics of behaviour change. The review by Sieck *et al*. ^64^ further indicates that educational and lifestyle interventions have more uncertain effects on measured chemical levels than policy measures and studies in which participants’ consumption is controlled. Moreover, in the two reviews cited, ^64^ ^,^ ^65^ interventions are not analyzed in terms of theoretical framework of behaviour change. Thus, only a limited number of intervention studies have specifically tested, based on a theoretical background, how to promote long-term, voluntary change of habits in relation to reducing EDCs exposure.

Studies focusing on knowledge and risk-awareness illustrate that it is not sufficient for inducing behaviour change. A first example is the PREVED -Pregnancy, PreVention, Endocrine Disruptors- study is an example of a workshop-based intervention study designed to reduce EDCs exposure in pregnant women, with a focus on canned food consumption. ^66^ The intervention focused on knowledge and risk perception, and the study sample size was 230, participants being for the majority born in Metropolitan France, and in a no precarious situation. Results showed that it failed to significantly change behaviour as in both control and intervention group there were similar proportion of participants whose consumption of canned food and drinks decreased, increased and remained stable. However, risk perception scores increased significantly in the intervention group. A second example is a study using biomonitoring as a technique to motivate exposure reduction. ^67^ The participating women (N = 135), nearly-half of them self-identified as Black/African American, received a personalized report mentioning either their individual chemical exposure levels, or exposure levels aggregated at the study level. Results were mixed: out of 15 behaviours, only four evolved in a positive direction (fewer food prepared using nonstick cookware; fewer microwave popcorn, and fewer use of a problematic dental floss; and more frequently having added or removed an item from the home due to concern about chemicals), the frequency of ten behaviours did not change, and one protective behaviour (damp-wiping dust) decreased at follow-up. Whether the biomonitoring report was individual or aggregated did not influence the results.

A few studies including techniques besides information provision showed positive effects on behaviour. The first one showed that an education program for Korean pregnant women, informing them about the effects of toxicants and providing strategies to practice protective behaviours, was successful in inducing behaviour change among participants exposed to the intervention (N = 40, compared to 56 in the control group), effect size Cohen’s *d* = 0.83 [ 1]. ^68^ However, a significant limitation of this study is the short timeframe of the evaluation: the post-test occurred four weeks after the intervention ended. Given the time it takes to develop new habits (range between 18 to 254 days according to Lally et al. ^69^), studies with longer time frames are needed to properly evaluate implementation of changes in daily lives. The study by Park and Chung, which targeted a cognitive bias that leads to consider oneself as less at risk than the average others, showed success in increasing protective behaviours in a sample of 140 young women. ^70^ The intervention group was exposed to several behaviour change techniques (BCTs), including self-checking one’s habits of exposure, an information session, assessing barriers to reduce exposure, and signing a health contract. Protective behaviours against EDCs included daily life habits such as consuming instant food from convenience stores and using disposable cups for example. The comparison of the evolution in the control group compared to the experimental group show an effect size of *d* = 1.29 [ 2].Their positive result is thus not surprising, as the intervention targeted a variety of psychological determinants and was not limited to providing knowledge. However, its quasi-experimental design and the lack of biological samples analyses reduce the reliability of these findings.

The mix-methods pilot study by Tsui et al. ^71^ provides a proof of methodological principle regarding an intervention developed with the community on the use of phthalate-free hair care products among pregnant Women of Color (mostly Hispanic, Black Hispanic, and Black non-Hispanic). The intervention, based on the Environmental Health Literacy framework ^72^ contained educational video, but also practical tips to reduce exposure. Tools to check product ingredient and alternative, less-toxic products were suggested to participants. The effect of the intervention was assessed through progression in the stages of change, and with urine samples collection. Qualitative data suggest a progression in the stages (moving from “never heard of EDCs, nor EDCs in hair care products” prior the intervention to “having decided to reduce their use of hair care products containing phthalates” and “having limited use of such products” after the intervention) and a reduction of phthalate metabolite concentrations.

A review by Martin *et al*. ^73^ of lifestyle interventions to reduce EDCs exposure among men and women in their reproductive age conclude on three key elements: 1) tailored education about how to avoid EDCs with interactive educational materials including question/answer option, 2) support and monitoring by counselors or peer discussion group to provide feedback and encouragement, 3) understand individual sources of exposure (through diaries for example) to provide replacement options for items known to contain EDCs.

## Example of an awareness-raising campaign in Belgium

As part of the Belgian National Action Plan on Endocrine Disruptors (NAPED, 2022–2026), aiming to raise awareness in vulnerable population groups (pregnant women, women planning pregnancy, young children (0–3 years), and adolescents), public awareness campaigns are planned. These campaigns are coordinated by the Federal Public Service Health, in close collaboration with regional and community authorities. It serves as an example of a public health intervention designed to increase awareness and knowledge of endocrine disruptors.

In May 2024, the first campaign, targeting pregnant women, was launched. It combined the delivery of practical, accessible advice with simple, everyday recommendations tailored to reduce exposure in daily life with a targeted digital communication strategy. Dissemination was based on strong partnerships and leveraged digital channels such as Meta, YouTube, Pinterest, and optimised web content through search engine optimisation.

Healthcare professionals were engaged during the campaign through an informative email, which invited them to download a specially developed Q&A document on EDCs. They were also given the opportunity to order promotional materials, such as postcards and posters, via the Federal Public Service website. In total, over 80,000 postcards and 16,500 posters were ordered and distributed to more than 1,300 healthcare professionals and institutions. A second campaign is planned for November–December 2025. It will broaden outreach to include caregivers of young children (0–3 years) while reinforcing key messages aimed at pregnant women and their partners.

An external evaluation, conducted by a polling institute before and after the campaign among a representative sample of 1,000 Belgians, revealed a generally positive reception, with an average satisfaction score of 7.3/10 overall, rising to 8.0/10 among pregnant women. Knowledge about EDCs increased by 7 percentage points, and the campaign generated widespread interest among both professionals and the general public.

However, despite these encouraging results, self-reported behavioural changes were limited. While participants expressed greater awareness, behavioural changes remained gradual. This highlights a common challenge in health promotion: while a one-off information campaign can effectively raise awareness and improve knowledge, it is rarely sufficient to produce sustained behaviour change.

## The HYPIEND behaviour change interventions

In the HYPIEND project, two multicomponent interventions have been designed with the aim of accompanying pregnant women and parents of prepubertal children to reduce their daily life exposure to EDCs. The interventions consist of a digital tool and a set of workshops. Moreover, a school-wide educational program based on explicit teaching of healthy behaviors and positive reinforcement and a plastic-free canteen will be implemented in the participating schools.

A reduction in EDCs exposure is expected through targeting the following categories of behaviour: Indoor air quality (e.g., ventilation, avoiding products with fragrances, choice of cleaning products, cleaning dust); choice of food (e.g., avoiding ultraprocessed food, fast-food, canned food and drinks and food with preservatives; favour organic when possible); food preparation (wash fruits and vegetables before eating and peel when relevant); food contact materials (to store, cook and consume); personal care products (choice of products, limit use of products); choice of clothing, furniture, toys and electronics; child’s behaviour (wash the baby/child’s hands after playing and before eating; for the perinatal study: do not allow to chew certain things; for the prepubertal study: have the child use a stainless steel bottle to drink); gardening and Do It Yourself activities; smoking; and occupational exposure (for the perinatal study only). Major targeted compounds are pesticides, bisphenols, PFAS, phthalates, parabens, benzophenones, flame retardants, PAHs, heavy metals, and isoflavones (the latter, for the prepubertal study only).

Both interventions rely on the Health Action Process Approach (HAPA). ^50^ This model was chosen because it is more comprehensive than alternative frameworks and does not treat intention as the sole predictor of behaviour. It clearly distinguishes between the motivational and volitional phases, emphasising how intentions are translated into action It is known that around one out of two decisions that individuals take will not be acted upon. ^74^ In the study by Harley et al., ^63^ the main reason for participants’ lack of compliance with the furnished low-chemical personal care products alternatives to theirs regular products was forgetting. This result highlights the importance of the volitional phase of behaviour change. For this reason, the HYPIEND interventions not only inform participants about what EDCs are and their potential impact but also inform them about changes that can be made at the individual level and contain techniques such as planning to help people implement their intentions. Those techniques are taken from a 93-techniques list, the BCTs Taxonomy. ^75^ The use of this taxonomy was dictated by the need of having an extensive choice of techniques to broadly cover behaviour change mechanisms. In addition, using techniques from the taxonomy allows to report the intervention ingredients in a standardized way, improving replicability for future interventions. ^75^ The appropriate techniques were selected using the Theory and Technique Tool, a related tool helping to identify relevant techniques based on empirical evidence. Participants from the control group will receive a booklet (also distributed to the intervention group) containing information about what EDCs are, and recommendations to reduce exposure, without any additional BCT.

Through the digital tool, participants are able to work on different missions aimed at minimizing exposure to EDCs. By selecting these missions successively, they receive frequent recommendations, tips and motivational quotes that help them to accomplish the selected mission. Some of the BCTs included in the digital tool are: graded tasks, used to increase task self-efficacy (i.e., initiate the change); action planning and problem solving, used to help intenders become actors; and providing tools to conserve mental resources, to increase maintenance self-efficacy (i.e., once the change starts and needs to become a habit).

**Figure 2.**
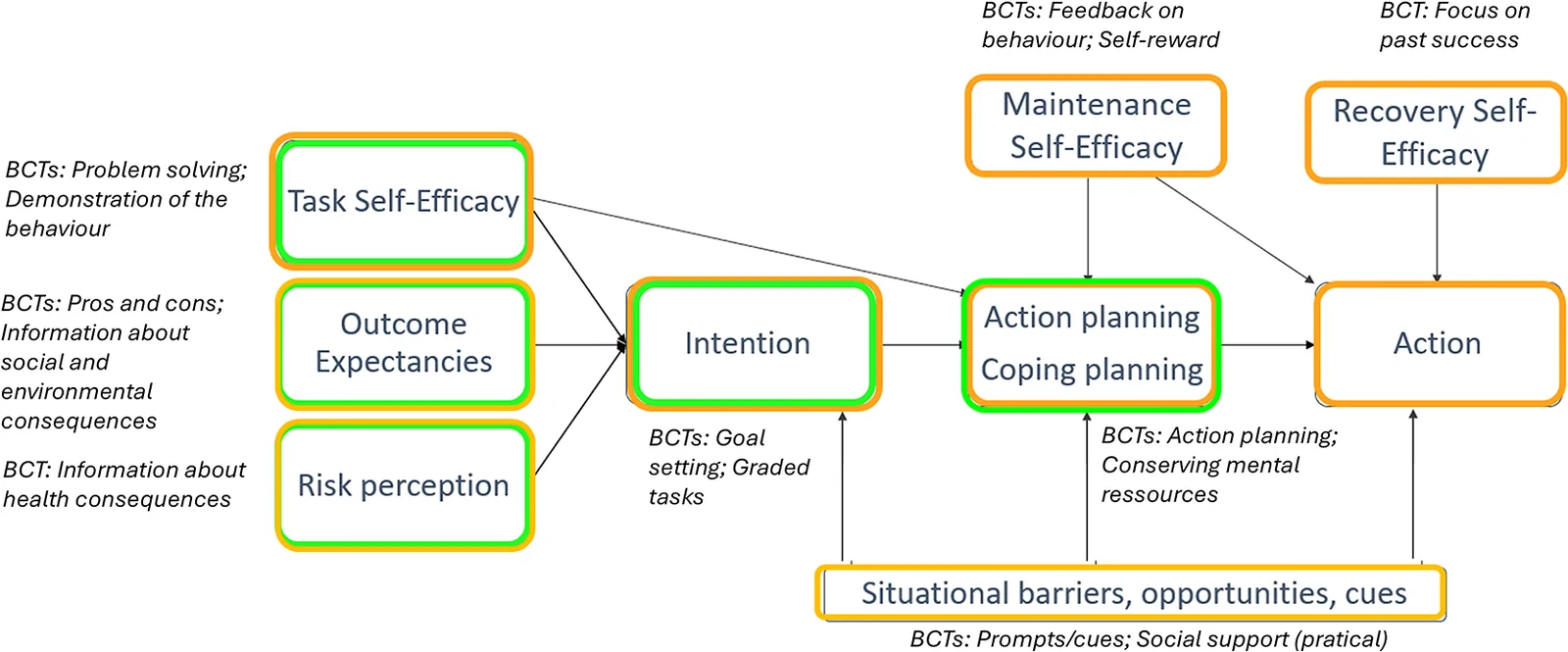
HAPA model, adapted from Schwarzer ^39^ to reflect which components of the model are targeted through which HYPIEND intervention aspect (workshops = green, digital tool = orange).

Another tool used in the HYPIEND interventions is motivational interviewing. ^76^ It is an interviewing strategy to help the person resolve ambivalence to change, and trigger motivation emerging from within, by letting the person themselves evoke the arguments instead of the counsellor providing them. In the workshops, the animator will provide a brief education about EDCs and spend most of the session using motivational interviewing techniques in group, to help participants build a strong motivation to change. Figure 2 illustrates how the digital tool and the workshops target the HAPA constructs, with examples of BCTs used for each construct. For the perinatal study, group size range between 2 and 8 participants, and maximum 25 parents for the prepubertal study.

Lifestyle interventions must consider the broader social–ecological context, including family dynamics. ^77^ For example, evidence shows that partner involvement in maternal health promotes healthier behaviours within households, ^78^ which is particularly relevant for reducing EDCs exposure, a process often requiring collective family action. ^73^ Yet, most interventions still focus solely on mothers. ^79^ Including partners in EDCs-related intervention studies is therefore essential for several reasons: it reflects the shared biological responsibility for reproductive health, it acknowledges the social and behavioural dynamics of households, and it allows interventions to target both partners to improve efficacy. Couple-based interventions are particularly well-suited for modifying shared behaviours, ^77^ such as food choices, product use, or environmental exposures at home, which are often difficult to change in isolation. For this reason, the HYPIEND intervention with pregnant women include the cohabiting partner, and the HYPIEND study on prepubertal children includes the whole family and the school through the School Wide Positive Behavioural Interventions and Supports (SWPBIS).

**Figure 3.**
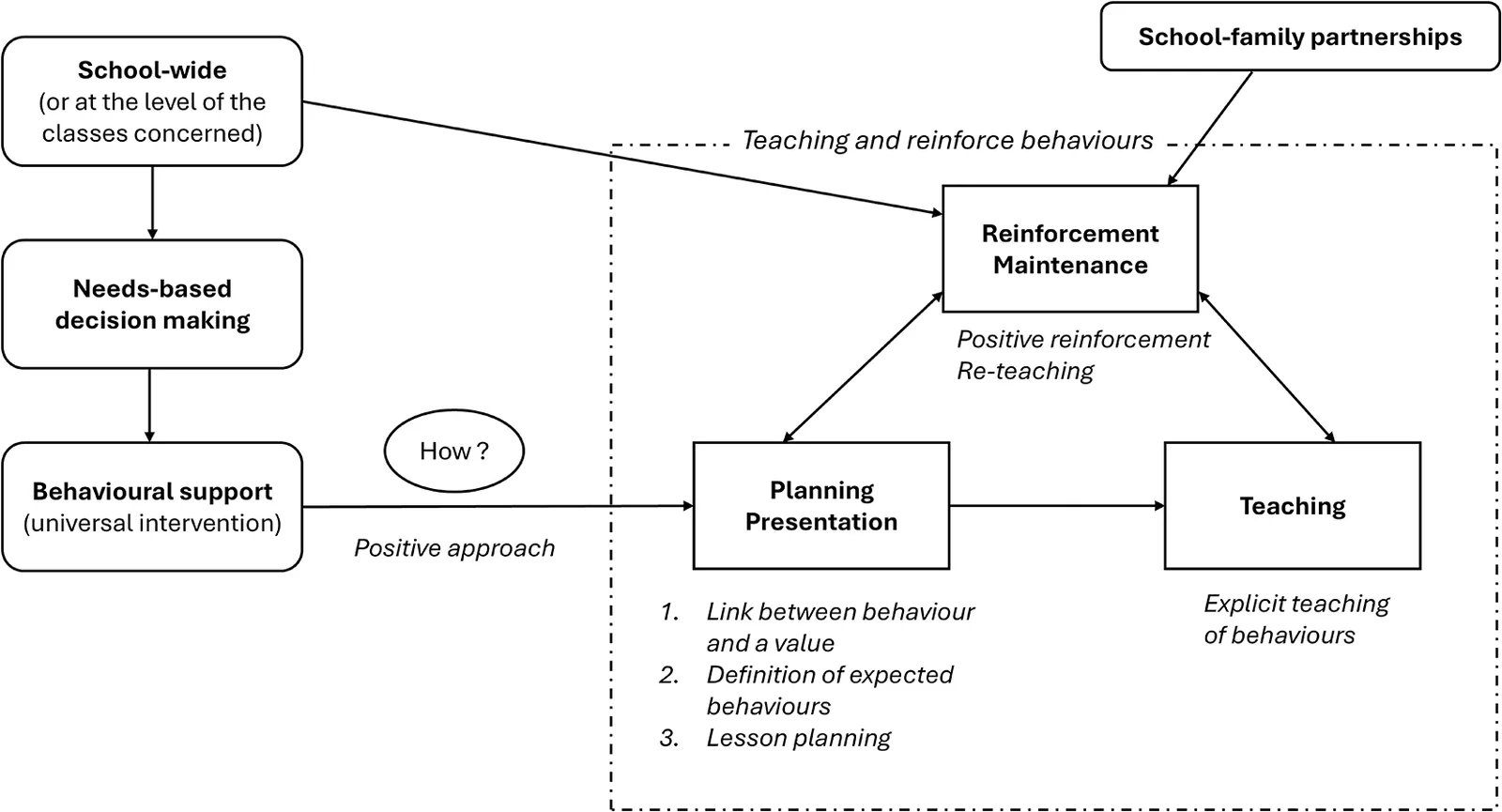
Model for supporting positive health behaviours among students as part of the HYPIEND project.

SWPBIS is a multi-tiered intervention designed to positively reinforce students’ expected behaviours at school. ^53^ It aims to create a positive and secure school climate by explicitly teaching and positively reinforcing expected behaviours. These are defined beforehand in observable terms and explicitly taught to students. SWPBIS insists on the sense of adopting a behaviour (“Why” is it important to adopt a specific behaviour at school) and it asks teachers to model the expected behaviour (“How” to perform this behaviour). It is implemented throughout the whole school by all adults supervising the children. The effects of SWPBIS have been documented in several systematic reviews. ^80^ ^–^ ^82^ Sugai and Horner’s ^53^ model of positive behaviour support has been revisited and adapted to the context of the HYPIEND project ( Figure 3).

Based on the needs and observations of school stakeholders, health-related behaviours will be identified and explicitly taught at the school level or within the classes involved in the project. The behaviours taught and promoted in schools for children are adapted to their autonomy level, and do not require any purchase (e.g. handwashing, ventilating rooms, washing fruit and vegetables, etc.). To support the adoption of these behaviours, strategies such as positive reinforcement and re-teaching sessions will be implemented. Reinforcement will be supported by the school-family partnership, the actions carried out at the whole-school level, and the planning and instructional phases. The positive reinforcement provided by the primary caregiver that we use in schools is likely to encourage the child to adopt and sustain the desired behaviours over time. Several theoretical frameworks can be drawn upon to explain this. According to the systematic review by Kwasnicka et al., ^83^ the maintenance of behaviours over time can be explained in particular either by changes in habits (behaviours which have become habitual and are supported by automatic responses to relevant cues) or by the social motivation provided by the social environment (a supportive environment and social support are important for the maintenance of behavioural change. People tend to maintain behaviour that is in line with relevant social changes). Finally, the work of Deci and Ryan ^84^ allows to formulate the hypothesis that social support (i.e. positive reinforcement from a significant adult in our case) facilitates the internalisation of motivation.

Taking actions to reduce EDCs exposure is not necessarily accessible in the same way to all socioeconomic groups. Some recommendations to avoid EDCs involve buying more costly alternatives (e.g. labelled products). However, in order to prevent generating a feeling of helplessness among participants with low-income, we emphasize solutions that can be adopted by everyone at no cost, such as ventilating the home, washing hands, use homemade all-purpose cleaners, or reducing the number of cosmetics products use. In order to avoid exacerbating inequities, it is important to consider the social determinants of health. ^85^ In the HYPIEND project, this means including the social factors such as the partner and the whole family, but also the environmental dimension through the school-level intervention. Notably, the Positive Behavioural Interventions and Supports (PBIS) program adapted in the HYPIEND project has a history of being used to improve learning and social outcomes for children in low-income families and neighbourhood. ^86^

The effectiveness of the HYPIEND interventions will be tested through a) a tricentric Randomized Controlled Trial (RCT) involving pregnant women and their infants up to 18 months of age in Spain, Belgium and Poland, and b) a bicentric RCT carried out at primary schools from Spain and Belgium for 28 months, targeted to prepubertal children and their parents. Through these RCTs we will evaluate the potential effectiveness of the multicomponent interventions to reduce the internal concentration of several EDCs families. We will further evaluate whether the anticipated reduction in exposure to EDCs can mitigate their potential adverse effects on growth, neurodevelopment, and pubertal maturation. To elucidate the biological mechanisms underlying EDCs-related health outcomes, we will conduct comprehensive analyses of hypothalamic-pituitary axis hormones, cytokines, epigenetic modifications (DNA methylation), gene expression profiles, and gut microbiota composition. Both studies include four measurement times. For the perinatal study the visits are planned at the following dates: V1 (pre-intervention) at 6–11 weeks of pregnancy, V2 at 28–32 weeks of pregnancy, V3 at 4–7 weeks post-delivery; V4 at 12 months post-delivery. For the prepubertal study, the visits are Month 1 (pre-intervention), Month 16, Month 22, Month 34. In both studies, urine, blood and feces are collected at V1 and V4. For the perinatal study, the offspring will be included in the measurements starting at V3. In terms of behaviour change, we will measure both the psychological determinants of behaviour using a HAPA questionnaire, and EDC-related lifestyles habits through a self-report questionnaire. We hypothesize that the intervention group will demonstrate an increase in the HAPA components, as well as a reduced exposure to EDCs. For the control group, we do not expect a significant difference overtime. Given the study design, it will not be possible to assess the effectiveness of each individual intervention component. For future work, the multiphase optimization strategy (MOST) offers a structured way to test ingredients. ^87^ The complete details on the RCTs protocols will be published shortly through specific papers, while the protocols for the perinatal and the prepubertal studies can already be found respectively here: https://clinicaltrials.gov/study/NCT07142447 and here https://clinicaltrials.gov/study/NCT07411001.

## Recommendations and conclusion

As stated by Davis *et al*., ^14^ understanding behaviour is necessary for interventions to have the greatest possible impact. However, despite this existing scientific knowledge about human behaviour, an idea remains among some health professionals and policy makers ^88^ ^–^ ^90^: ignorance is the problem, and education a straightforward solution. ^89^ Simis *et al*. mentions in their essay that an information deficit model persists because of the “lure of rationality” ^89^ ^p.400^ - the appealing but flawed notion that humans are primarily rational beings who will make logical choices once they are properly informed. This idea is attractive because it simplifies complex behavioural issues into a solvable knowledge gap, allowing experts to assume that mere education alone can change behaviour. However, as mentioned above, human decision-making is influenced by a range of psychological and social factors that go beyond rational analysis.

According to Lippke and Ziegelmann, several reviews and meta-analyses showed that a majority of behavioural interventions reported no theoretical background. ^91^ We support the call in advocating an improvement in the quality of behaviour change interventions by relying on evidence-based models, ^14^ ^,^ ^91^ and advocate that this should also be the case for the field of EDCs.

This paper illustrates a research project mobilizing behaviour change techniques to reduce EDCs exposure at the individual level. However, this should not be considered as putting the responsibility on individuals themselves. On the contrary, the message is to urge the scientific community, health professionals and policy-makers to make better public health interventions on this topic. In addition to communication, other policy categories are also essential to have a stronger and lasting impact, such as legislation, service provision and regulation. ^92^ The ENKORE [ 3] policy brief ^93^ calls for actions such as revisions of relevant EU regulation for a better protection of consumers and moving beyond chemical-by-chemical assessment approach to better reflect real-life exposure. In this context, behavioural approach could be useful to improve the impact of structural measures: for example. Lentz et al. showed that support for public policies such as meat taxes, plant-based food subsidies, and increasing the availability of plant-based meal in public institutions could be explained by respondents’ beliefs. ^94^ Given that political will often depends on public support, ^95^ it is important to consider individual-level perspective even for structural measures.

To conclude, given the important health and environmental adverse effects of these substances, any attempt to change behaviours regarding exposure to EDCs should be carefully crafted to increase the chances of maximum efficacy. So that EDCs-related interventions have the best possible chance on being effective both biologically and behaviourally, they must be tailored and holistic, actively involve the broader social environment and go beyond education and awareness by incorporating concrete and evidence-based BCTs. These elements are crucial to enhance motivation, engagement, and adherence, ultimately maximize the probability of improving outcomes in both research and practice.

## Ethics and consent

Ethical approval and consent were not required.

## Data availability

### Extended data

Appendix are uploaded on Zenodo.

DOI Figure S1: 10.5281/zenodo.21025393.

DOI Figure S2: 10.5281/zenodo.21025855.

## Footnotes

^1^
Effect size calculated using https://www.psychometrica.de/effect_size.html based on the means and standard deviation reported in the paper.

^2^
Effect size calculated using https://www.psychometrica.de/effect_size.html based on the means and standard deviation reported in the paper.

^3^
The ENKORE cluster is a group of 5 research projects (including HYPIEND) funded under the same call “Health impacts of endocrine-disrupting chemicals: bridging science-policy gaps by addressing persistent scientific uncertainties”.
