# Supplementary material for: Using evidence-based models of human behaviour to reduce endocrine-disrupting chemicals exposure: recommendations for strengthening interventions
Source: Open Res Eur. 2026 Jul 16;5:385. Originally published 2025 Dec 12. [Version 2] doi: 10.12688/openreseurope.21765.2 (PMC13555062; doi:10.12688/openreseurope.21765.2)
Supplement: Peer review history

# Reviewer report: Reviewer response for version 2

**DOI:** 10.21956/openreseurope.25567.r77482

**Published:** 2026-08-10

**Authors:** Dede Teteh-Brooks

**Affiliations:** 1 UT MD Anderson Cancer Center, Houston, Texas, USA

Bravo to the authors for the improvements made on the manuscript. The revisions are appropriate and I approve this version for indexing. Best wishes on your implementation efforts.

Where applicable, are recommendations and next steps explained clearly for others to follow? (Please consider whether others in the research community would be able to implement guidelines or recommendations and/or constructively engage in the debate)

Partly

Does the article adequately reference differing views and opinions?

Partly

Are all factual statements correct, and are statements and arguments made adequately supported by citations?

Partly

Is the rationale for the Open Letter provided in sufficient detail? (Please consider whether existing challenges in the field are outlined clearly and whether the purpose of the letter is explained)

Yes

Is the Open Letter written in accessible language? (Please consider whether all subject-specific terms, concepts and abbreviations are explained)

Yes

Reviewer Expertise:

Health disparities, equity informed community-based intervention, environmental exposures in personal care products, CBPR

I confirm that I have read this submission and believe that I have an appropriate level of expertise to confirm that it is of an acceptable scientific standard.

# Reviewer report: Reviewer response for version 2

**DOI:** 10.21956/openreseurope.25567.r77480

**Published:** 2026-07-27

**Authors:** Jasmine McDonald

**Affiliations:** 1 Columbia University Irving Medical Center, New York, New York, USA

The authors did a great job in responding to my comments. I have no further comments with the exception of the below. I vote to Accept the manuscript for indexing.

 Re: #6. Discuss a successful cohabitation intervention? 

 Answer:  we are not familiar with “cohabitation intervention”, could you please clarify what you mean by that?

 I was speaking about those living in the same household being impacted by a chemical exposure reduction intervention.

Where applicable, are recommendations and next steps explained clearly for others to follow? (Please consider whether others in the research community would be able to implement guidelines or recommendations and/or constructively engage in the debate)

Yes

Does the article adequately reference differing views and opinions?

Yes

Are all factual statements correct, and are statements and arguments made adequately supported by citations?

Yes

Is the rationale for the Open Letter provided in sufficient detail? (Please consider whether existing challenges in the field are outlined clearly and whether the purpose of the letter is explained)

Yes

Is the Open Letter written in accessible language? (Please consider whether all subject-specific terms, concepts and abbreviations are explained)

Yes

Reviewer Expertise:

Breast Cancer; Molecular Epidemiology; Environmental Exposures

I confirm that I have read this submission and believe that I have an appropriate level of expertise to confirm that it is of an acceptable scientific standard.

# Reviewer report: Reviewer response for version 2

**DOI:** 10.21956/openreseurope.25567.r77483

**Published:** 2026-07-24

**Authors:** Moses O Alfred, Oyedolapo Bamigboye

**Affiliations:** 1 Redeemer’s University, Ede, Nigeria; 2 Ilishan-Remo, Babcock University School of Basic and Applied Sciences (Ringgold ID: 309474), Ede, Ogun State, Nigeria

Authors have sufficiently addressed all issues raised in the revised document. The manuscript is significantly improved for indexing.

Where applicable, are recommendations and next steps explained clearly for others to follow? (Please consider whether others in the research community would be able to implement guidelines or recommendations and/or constructively engage in the debate)

Yes

Does the article adequately reference differing views and opinions?

Yes

Are all factual statements correct, and are statements and arguments made adequately supported by citations?

Partly

Is the rationale for the Open Letter provided in sufficient detail? (Please consider whether existing challenges in the field are outlined clearly and whether the purpose of the letter is explained)

Yes

Is the Open Letter written in accessible language? (Please consider whether all subject-specific terms, concepts and abbreviations are explained)

Yes

Reviewer Expertise:

Water Quality Monitoring; Water Treatment; Evidence-based policy engagement; Environmental quality monitoring; CO2 conversion; climate

We confirm that we have read this submission and believe that we have an appropriate level of expertise to confirm that it is of an acceptable scientific standard.

# Reviewer report: Reviewer response for version 2

**DOI:** 10.21956/openreseurope.25567.r77481

**Published:** 2026-07-24

**Authors:** Robb Shawe

**Affiliations:** 1 Capitol Technology University,, Laurel, Maryland, USA

I have reviewed the revisions and determined that the author has successfully adhered to the recommendations provided.

 V/r

 Dr. Robb Shawe

Where applicable, are recommendations and next steps explained clearly for others to follow? (Please consider whether others in the research community would be able to implement guidelines or recommendations and/or constructively engage in the debate)

Yes

Does the article adequately reference differing views and opinions?

Yes

Are all factual statements correct, and are statements and arguments made adequately supported by citations?

Partly

Is the rationale for the Open Letter provided in sufficient detail? (Please consider whether existing challenges in the field are outlined clearly and whether the purpose of the letter is explained)

Yes

Is the Open Letter written in accessible language? (Please consider whether all subject-specific terms, concepts and abbreviations are explained)

Yes

Reviewer Expertise:

Environmental Health & Exposure Science, Behaviour Change Science & Health Psychology, Public Health Intervention Design, Risk Communication & Health Communication, Systems Thinking & Social‑Ecological Models, and Occupational and Environmental Safety Regulation

I confirm that I have read this submission and believe that I have an appropriate level of expertise to confirm that it is of an acceptable scientific standard.

# Reviewer report: Reviewer response for version 1

**DOI:** 10.21956/openreseurope.23544.r68703

**Published:** 2026-02-28

**Authors:** Jasmine McDonald

**Affiliations:** 1 Columbia University Irving Medical Center, New York, New York, USA

The article by Moussaoui and colleagues describes the current interventions involving pregnant women and interventions involving pubertal children. The authors then go on to describe their own intervention, HYPIEND - a multilevel intervention for pregnant women and children designed using the HAPA and Theory and Technique behavioral tool. I have several comments to strengthen the papers utility to the environmental health and intervention community:

 Introduction: 

 1. Given pregnant persons and children will be discussed, the opening paragraph regarding routes of exposure should include breast milk and placenta.

 2. Re: Lifestyle interventions: Are these all relevant studies for pregnant women and children?

 - The author should detail the demographics of the articles discussed/included given the Open Letter's focus on vulnerable populations.

 - Overall, greater descriptions of studies and outcomes (including magnitude) would be helpful; for example, the PREVED and report-back study could use more detail. For example, how many women in PREVED, what were the four behaviors evolving into a positive direction and is this lesson learned incorporated into HYPIEND? Do the authors deem report-back unimportant thus not included in HYPIEND and what/how/when was information reported back relative to change assessment?

 - There is also the NY-based studies in pregnant women that is not mentioned (citation below).

 - Moreover, depending on the age cut-off for pubertal children, there is also the HERMOSA study (citation below).

 - Discuss a successful cohabitation intervention?

 Model Clarity

 Within the discussion of evidence based models, the models used in past studies are not named - or are they non-existent. Limitations are noted, but not the theory of behavior applied to invoke change. 

 Study Length

 - Authors note a limitation in past studies in the length of time. While I agree sustainable behaviors require a longer view - what is long? Many EDCs have short half-lives and change can be observed within days - especially for phthalates (please see the HERMOSA study among adolescents). How do authors define long-term behavioral or the formation of a habit?

 HYPIEND Study Design

 - The HYPIEND project fails to describe action steps if replacement items are not affordable or unavailable.

 - Figure 2, does this model translate for pregnant women and children?

 - Figure 3, how does child-based/school-based behavioral maintenance occur? Through what autonomy? Many children do not have purchasing or selection autonomy; therefore, how will efficacy be promoted in SWPBIS?

 - How large should groups be during motivational interviewing? Are children involved?

 - Greater detail on EDC-related biological health outcomes and source/medium used to assess - urine, blood, from whom? frequency? pre-/post-?

Where applicable, are recommendations and next steps explained clearly for others to follow? (Please consider whether others in the research community would be able to implement guidelines or recommendations and/or constructively engage in the debate)

Yes

Does the article adequately reference differing views and opinions?

Yes

Are all factual statements correct, and are statements and arguments made adequately supported by citations?

Yes

Is the rationale for the Open Letter provided in sufficient detail? (Please consider whether existing challenges in the field are outlined clearly and whether the purpose of the letter is explained)

Yes

Is the Open Letter written in accessible language? (Please consider whether all subject-specific terms, concepts and abbreviations are explained)

Yes

Reviewer Expertise:

Breast Cancer; Molecular Epidemiology; Environmental Exposures

I confirm that I have read this submission and believe that I have an appropriate level of expertise to confirm that it is of an acceptable scientific standard, however I have significant reservations, as outlined above.

## References

[1] : Reducing Phthalate, Paraben, and Phenol Exposure from Personal Care Products in Adolescent Girls: Findings from the HERMOSA Intervention Study. *Environmental Health Perspectives* .2016;124(10) : 10.1289/ehp.15105141600-1607 10.1289/ehp.1510514 PMC504779126947464

[2] : Hair care product use among pregnant women of color: protocol for a feasibility educational intervention. *Frontiers in Reproductive Health* .2026;7: 10.3389/frph.2025.1694088 10.3389/frph.2025.1694088 PMC1287287641659359

[3] : The hair tales of women of color in Northern Manhattan: a qualitative analysis. *Frontiers in Reproductive Health* .2024;6: 10.3389/frph.2024.1298615 10.3389/frph.2024.1298615 PMC1097879838559324

# Response

**Published:** 2026-04-07

**Authors:** Lisa Moussaoui

**Affiliations:** Section de Psychologie, Universite de Geneve Faculte de Psychologie et des Sciences de l'Education, Geneva, Geneva, Switzerland

**We thank the reviewer for the valuable comments and explain below how we addressed each of them. **  

 The article by Moussaoui and colleagues describes the current interventions involving pregnant women and interventions involving pubertal children. The authors then go on to describe their own intervention, HYPIEND - a multilevel intervention for pregnant women and children designed using the HAPA and Theory and Technique behavioral tool. I have several comments to strengthen the papers utility to the environmental health and intervention community: 

 Introduction:  

**#1.** Given pregnant persons and children will be discussed, the opening paragraph regarding routes of exposure should include breast milk and placenta. 

**Answer: **Thank you for this relevant comment, we now mention breast milk and placenta as routes of exposure p.6 l.51-53.   

**#2. **Re: Lifestyle interventions: Are these all relevant studies for pregnant women and children? 

 - The author should detail the demographics of the articles discussed/included given the Open Letter's focus on vulnerable populations. 

**Answer: **Not all studies cited included pregnant women or children participants, however given the scarcity of studies on the topic we decided to cite even interventional studies that included non-vulnerable population. We now have added more demographics details to the study we cite when the information is available (for PREVED:, p.8 l.155-157; Boronow et al,. study: p.8 l.161-163, Kim et Jeong study: p.9 l.172, Tsui et al. p.9 l.194-195). For the study by Park and Chung, no details were provided on the population besides being young women.    

**#3.** Overall, greater descriptions of studies and outcomes (including magnitude) would be helpful; for example, the PREVED and report-back study could use more detail. For example, how many women in PREVED, what were the four behaviors evolving into a positive direction and is this lesson learned incorporated into HYPIEND? Do the authors deem report-back unimportant thus not included in HYPIEND and what/how/when was information reported back relative to change assessment? 

**Answer: **To answer this comment we calculated effect sizes (cohen’s d, based on means and SD reported in the papers) in order to give the reader a better estimate of the outcome's magnitude (p.9 l.175-176, and p.9 l.187-188). The sample sizes were also included. Regarding the Boronow et al. study, we now specify in the text the four behaviours that evolved in the positive direction (p.8 l.165-l.167). This result was not directly incorporated into HYPIEND given that the intervention techniques were very different. We did not include report-back in our intervention because this specific technique did not emerge in our intervention conception logic (using the HAPA constructs and then using the theory and technique tool). This does not mean we judge the technique unimportant. It is the same as for comparative optimism in Park and Chung study, that resulted in a positive outcome, but we did not include it because it did not fit our theoretical framework. In addition to the logic of the intervention construction, we were also very careful to avoid triggering too much anxiety in our participants, both from vulnerable groups. Biofeedback might trigger anxiety, and the effect of fear on behaviour change has been debated a lot in the literature (see e.g. Kok et al. 2017, https://doi.org/10.1080/17437199.2017.1415767).      

**#4. **There is also the NY-based studies in pregnant women that is not mentioned (citation below). 

**Answer: **Thank you for informing us about this recent publication, we added it in the text p.9 l.192-203.   

**#5.** Moreover, depending on the age cut-off for pubertal children, there is also the HERMOSA study (citation below). 

**Answer: ** the cut-off age for prepubertal children is lower, but we included the HERMOSA study in the paper as it provides an example of controlled consumption intervention study for personal care products and the reduction of chemicals in urine (p.8 l.135-13), and also highlight the challenges to behaviour change even when the products are provided (p.11 l.267-270).   

**#6.** Discuss a successful cohabitation intervention? 

**Answer: ** we are not familiar with “cohabitation intervention”, could you please clarify what you mean by that?    

**#7. **Model Clarity 

 Within the discussion of evidence based models, the models used in past studies are not named - or are they non-existent. Limitations are noted, but not the theory of behavior applied to invoke change.  

**Answer: ** we purposefully did not mention names of the theoretical models in the section “evidence-based models of behaviour change” following a reviewer’s comment in a previous round that it was “the alphabet soup of the behavioral change models”, and decided to focus on the theoretical concepts rather than the theories themselves to make this part more accessible to a broad readership. Because the theories themselves were not introduced in this section, we decided to not mention them later in the paper as they were not previously defined.    

**#8. **Study Length 

 - Authors note a limitation in past studies in the length of time. While I agree sustainable behaviors require a longer view - what is long? Many EDCs have short half-lives and change can be observed within days - especially for phthalates (please see the HERMOSA study among adolescents). How do authors define long-term behavioral or the formation of a habit? **Answer: **Our study focus is not on the immediate observation of changes in biological samples responding to change of products as it has been done in other studies, but rather in the feasibility for families to implement real change of everyday behaviours in an uncontrolled context (e.g. without replacement products provided.) For this reason, we rely on the work by Lally and al. to define the length of habit formation (range between 18-254 days). We now explicit this length in the text p.9 l.178.     

**#9. **HYPIEND Study Design 

 - The HYPIEND project fails to describe action steps if replacement items are not affordable or unavailable. 

**Answer: ** no replacement items are provided in the HYPIEND study. However, in the intervention materials, especially the digital tool messages, there are tips for participants on how to solve those barriers of price or availability. Those tips belong to the problem solving behaviour change technique (mentioned p.11 l.285). The exact content of the messages will be the topic of another publication. 

**#10. ** Figure 2, does this model translate for pregnant women and children? 

**Answer: ** figure 2 aims to illustrate the theoretical model used to build the intervention. However, the translation for participant is presented in the theory of change tables that are now present in appendix   

**#11. ** Figure 3, how does child-based/school-based behavioral maintenance occur? Through what autonomy? Many children do not have purchasing or selection autonomy; therefore, how will efficacy be promoted in SWPBIS? 

**Answer: ** The child has no choice in school indeed, as it is the teachers who consult with one another to select behaviours based on feasibility, the needs in a particular school and our recommendations. That said, the positive reinforcement provided by the primary caregiver that we use in schools is likely to encourage the child to adopt and sustain the desired behaviours over time. Several theoretical frameworks can be drawn upon to explain this. According to the systematic review by Kwasnicka et al. (2016), the maintenance of behaviours over time can be explained in particular either by changes in habits (behaviours which have become habitual and are supported by automatic responses to relevant cues) or by the social motivation provided by the social environment (a supportive environment and social support are important for the maintenance of behavioural change. People tend to maintain behaviour that is in line with relevant social changes). Finally, the work of Deci and Ryan (2000) allows to formulate the hypothesis that social support (i.e. positive reinforcement from a significant adult in our case) facilitates the internalisation of motivation.  With regard to purchases, the behaviours taught and promoted in schools for children do not require any purchase (e.g. handwashing, ventilating rooms, washing fruit and vegetables, etc.).  We now mention this in the text p.13 l. 331-333 and l.336-346.     

**#12. ** How large should groups be during motivational interviewing? Are children involved? **Answer: **Children are not involved in the workshops. For the prepubertal study the workshops take place in the evening at school, similarly to regular school-parents meeting. Group size vary a lot depending on the number of parents participating in the study in one school, the maximum is 25 parents.  For the perinatal study, groups range between 2 and 8 participants and depends on the number of participants at that time in the intervention arm and available for that specific workshop. We do not have any theory-based recommendation on how large groups should be.  The range of group size is now mentioned p.12 l.297-298   

**#13. ** Greater detail on EDC-related biological health outcomes and source/medium used to assess - urine, blood, from whom? frequency? pre-/post-? 

**Answer:**  We now briefly describe EDC-related biological outcomes p.13-14 l.370-375. Given that the aim of the paper is to provide a conceptual commentary on behaviour change interventions, and that there will be separate publications for the study protocols, we limited the provided information to a summary of the measurement points and main outcomes.

# Reviewer report: Reviewer response for version 1

**DOI:** 10.21956/openreseurope.23544.r68999

**Published:** 2026-02-19

**Authors:** Dede Teteh-Brooks

**Affiliations:** 1 UT MD Anderson Cancer Center, Houston, Texas, USA

**Summary of review:**

 This manuscript addresses an important challenge in EDC exposure reduction by advocating for evidence-based behavior change models rather than information-only approaches. The HYPIEND and SWPBIS project may potentially support vulnerable stakeholders dwelling in European and other countries given the dearth of behavioral interventions available. However, the paper has significant clarity and presentation issues that make it difficult to follow and understand the interventions being proposed.

**Key Issues:**

- Several paragraphs are lacking references to support statements. Include references to support key claims throughout the manuscript.
  - For example: statements about EDCs representing 'a threat for the ecosystem and human health' (page 1), claims that 'current attempts to reduce modifiable exposure often rely on knowledge provision and awareness-raising campaigns' (page 1), and assertions about the transition from awareness to behavior change not being 'straightforward' (page 1) all need supporting references.
- Unclear manuscript identity and purpose. As discussed by previous reviewers the inclusion of a narrative literature review, conceptual framework and protocol description is creating confusion on the intended primary purpose of the manuscript.
  - Recommendation: Shorten the narrative review and provide a protocol description of the two interventions
- Intervention components and distinctions are unclear. The introduction of the two interventions with their components are not distinct in the current presentation.
  - Recommendation: Provide a summary table of each components side-by-side.
  - Suggested narrative structure **; **for each intervention, present separately: Introduction of the interventions; Development process including theories, models, and frameworks; study design - protocol
- Evaluation of implementation components. The manuscript describes multicomponent behavioral interventions but does not specify which implementation strategies or frameworks will be used to test and evaluate the distinct components, making it unclear how the effectiveness of individual intervention elements will be assessed.
- Highlighting two comments from previous reviewers that should be included in manuscript revisions:
  - Organization and presentation of narrative review, framework, and protocol
  - Equity and accessibility considerations: will the populations with highest EDC exposures benefit from these interventions?

**Overall Assessment**:

 While this paper addresses an important topic, the current presentation makes it difficult to understand the interventions and their relationships. Clearer organization, better headings, and comprehensive referencing are needed to make this work accessible and impactful.

Where applicable, are recommendations and next steps explained clearly for others to follow? (Please consider whether others in the research community would be able to implement guidelines or recommendations and/or constructively engage in the debate)

Partly

Does the article adequately reference differing views and opinions?

Partly

Are all factual statements correct, and are statements and arguments made adequately supported by citations?

Partly

Is the rationale for the Open Letter provided in sufficient detail? (Please consider whether existing challenges in the field are outlined clearly and whether the purpose of the letter is explained)

Yes

Is the Open Letter written in accessible language? (Please consider whether all subject-specific terms, concepts and abbreviations are explained)

Yes

Reviewer Expertise:

Health disparities, equity informed community-based intervention, environmental exposures in personal care products, CBPR

I confirm that I have read this submission and believe that I have an appropriate level of expertise to confirm that it is of an acceptable scientific standard, however I have significant reservations, as outlined above.

# Response

**Published:** 2026-04-07

**Authors:** Lisa Moussaoui

**Affiliations:** Section de Psychologie, Universite de Geneve Faculte de Psychologie et des Sciences de l'Education, Geneva, Geneva, Switzerland

We thank the reviewer for the valuable comments and explain below how we addressed each of them.   

**Summary of review:**

 This manuscript addresses an important challenge in EDC exposure reduction by advocating for evidence-based behavior change models rather than information-only approaches. The HYPIEND and SWPBIS project may potentially support vulnerable stakeholders dwelling in European and other countries given the dearth of behavioral interventions available. However, the paper has significant clarity and presentation issues that make it difficult to follow and understand the interventions being proposed.  

**Key Issues:** **Reviewer's comment #1: **Several paragraphs are lacking references to support statements. Include references to support key claims throughout the manuscript. For example: statements about EDCs representing 'a threat for the ecosystem and human health' (page 1), claims that 'current attempts to reduce modifiable exposure often rely on knowledge provision and awareness-raising campaigns' (page 1), and assertions about the transition from awareness to behavior change not being 'straightforward' (page 1) all need supporting references. 

**Answer: **We have added references to support those sentences (for “statements about EDCs representing 'a threat for the ecosystem and human health” p.5 l.17 and for “assertions about the transition from awareness to behavior change not being 'straightforward'” p.5 l.34). For the claim that “current attempts to reduce modifiable exposure often rely on knowledge provision and awareness-raising”, to our knowledge there is no scientific publication stating this. This sentence is an observation from our reading of the literature and also professional experience with campaigns such as the example in Belgium described in the paper. We acknowledge that this might be an insufficient answer to the comment and added a sentence and a reference to illustrate how information and awareness-raising is argued as being the solution to change individuals' exposure to EDCs (p.5 l.28-33). 

**Reviewer's comment #2: **Unclear manuscript identity and purpose. As discussed by previous reviewers the inclusion of a narrative literature review, conceptual framework and protocol description is creating confusion on the intended primary purpose of the manuscript.  Recommendation: Shorten the narrative review and provide a protocol description of the two interventions 

**Answer: **We thank you for the recommendation. We do not feel that shorten the narrative review would be appropriate because we believe it’s an important part of the open letter. However, to address this comment and similar ones from other reviewers, we clarified the identity of the manuscript (p.5-6 l.42-l.46). The protocol of the interventions are the object of separate publication, however in order to clarify the content of the intervention we added a theory of change in appendix 

**Reviewer's comment #3: **Intervention components and distinctions are unclear. The introduction of the two interventions with their components are not distinct in the current presentation.  Recommendation: Provide a summary table of each components side-by-side.  Suggested narrative structure; for each intervention, present separately: Introduction of the interventions; Development process including theories, models, and frameworks; study design – protocol 

**Answer: **We followed the recommendation to include a theory of change of the interventions, and created one for each intervention to make it clearer between perinatal and prepubertal (see appendix)   

**Reviewer's comment #4: **Evaluation of implementation components. The manuscript describes multicomponent behavioral interventions but does not specify which implementation strategies or frameworks will be used to test and evaluate the distinct components, making it unclear how the effectiveness of individual intervention elements will be assessed. 

**Answer: **The added theory of change schema aims to clarify the content of the intervention. Regarding the test of the individual intervention elements, unfortunately the study design will not allow such evaluation, given we have only one intervention group exposed to the intervention packaged, compared to the control group. We now mention this as a limitation and refer to multiphase optimization strategy (MOST) for future studies p.14 l.380-382.  Highlighting two comments from previous reviewers that should be included in manuscript revisions: 

**Reviewer's comment #5: **Organization and presentation of narrative review, framework, and protocol 

**Answer: **We clarified the aim of the paper (an opinion letter), theory of change, and refer to protocol papers. 

**Reviewer's comment #6: **Equity and accessibility considerations: will the populations with highest EDC exposures benefit from these interventions? 

**Answer: **We included a discussion on equity p.13 l.347-358. 

# Reviewer report: Reviewer response for version 1

**DOI:** 10.21956/openreseurope.23544.r68993

**Published:** 2026-02-19

**Authors:** Robb Shawe

**Affiliations:** 1 Capitol Technology University,, Laurel, Maryland, USA

**Full Reviewer Report**

 The manuscript addresses an important gap in environmental health intervention design by integrating behavioural science into EDC exposure‑reduction strategies. The authors successfully demonstrate that knowledge-only approaches are insufficient and provide a compelling rationale for multicomponent, theory-driven interventions.

**Strengths**

- Clear articulation of the problem and rationale.
- Comprehensive review of behavioural models.
- Balanced discussion of successful and unsuccessful interventions.
- Strong justification for including partners, families, and schools.
- Well-structured presentation of HYPIEND as an illustrative model.

**Major Points Requiring Revision**

1. **Clarify the manuscript's primary contribution.**
  Explicitly state that this is a conceptual/methodological Open Letter, not an empirical evaluation.
2. **Avoid implying the demonstrated effectiveness of HYPIEND.**
  Use conditional or future-oriented phrasing consistently.
3. **Provide a brief justification for selecting HAPA and BCT Taxonomy** over other behavioural frameworks.
4. **Strengthen the causal logic of the intervention.**
  A theory‑of‑change table or paragraph would improve clarity.
5. **Address equity considerations.**
  Discuss how socio-economic constraints may affect the feasibility of recommended behaviours.
6. **Clarify that behavioural interventions complement—not replace—policy and regulatory actions.**

**Minor Points**

- Expand summaries of key studies to include population and scope.
- Correct typographical errors ("informationsession", "unclearer effects").
- Consider adding an appendix listing key EDCs referenced.
- Ensure global—not only EU—context is acknowledged.

**Conclusion**

 The manuscript is scientifically sound and makes a valuable contribution. With the revisions outlined above—particularly clarifying the manuscript's purpose, strengthening the causal logic, and refining the language regarding expected outcomes—it will be well-positioned for indexing.

Where applicable, are recommendations and next steps explained clearly for others to follow? (Please consider whether others in the research community would be able to implement guidelines or recommendations and/or constructively engage in the debate)

Yes

Does the article adequately reference differing views and opinions?

Yes

Are all factual statements correct, and are statements and arguments made adequately supported by citations?

Partly

Is the rationale for the Open Letter provided in sufficient detail? (Please consider whether existing challenges in the field are outlined clearly and whether the purpose of the letter is explained)

Yes

Is the Open Letter written in accessible language? (Please consider whether all subject-specific terms, concepts and abbreviations are explained)

Yes

Reviewer Expertise:

Environmental Health & Exposure Science, Behaviour Change Science & Health Psychology, Public Health Intervention Design, Risk Communication & Health Communication, Systems Thinking & Social‑Ecological Models, and Occupational and Environmental Safety Regulation

I confirm that I have read this submission and believe that I have an appropriate level of expertise to confirm that it is of an acceptable scientific standard, however I have significant reservations, as outlined above.

# Response

**Published:** 2026-04-07

**Authors:** Lisa Moussaoui

**Affiliations:** Section de Psychologie, Universite de Geneve Faculte de Psychologie et des Sciences de l'Education, Geneva, Geneva, Switzerland

The manuscript addresses an important gap in environmental health intervention design by integrating behavioural science into EDC exposure‑reduction strategies. The authors successfully demonstrate that knowledge-only approaches are insufficient and provide a compelling rationale for multicomponent, theory-driven interventions.   

 Strengths  

- Clear articulation of the problem and rationale. 

- Comprehensive review of behavioural models. 

- Balanced discussion of successful and unsuccessful interventions. 

- Strong justification for including partners, families, and schools. 

- Well-structured presentation of HYPIEND as an illustrative model. 

  Major Points Requiring Revision  

1. Clarify the manuscript's primary contribution. 

  Explicitly state that this is a conceptual/methodological Open Letter, not an empirical evaluation. 

 Answer: we agree that this was unclear and now explicitly state the main contribution of the paper p.5-6 l.44-46. 

1. Avoid implying the demonstrated effectiveness of HYPIEND. 

  Use conditional or future-oriented phrasing consistently. 

 Answer: we reformulated the sentences that were confusing about the demonstrated effectiveness (p.5 l.36; p.10 l.242-243; l.249; p.13 l.363-364;  p.15 l.420-421, l.425)  

1. Provide a brief justification for selecting HAPA and BCT Taxonomy over other behavioural frameworks. 

Answer:  we added justification for the choice of the HAPA (p.11 l.262-266 and l.267-270) and  BCT taxonomy  (p.11 l.274-278)   

1. Strengthen the causal logic of the intervention. 

  A theory‑of‑change table or paragraph would improve clarity. 

 Answer: A theory of change is now present in appendix. 

 Address equity considerations.    Discuss how socio-economic constraints may affect the feasibility of recommended behaviours. 

 Answer: we added a section on socio-economic inequities p.13 l.347-358. 

 Clarify that behavioural interventions complement—not replace—policy and regulatory actions. 

 Answer: we clarified p.14 l.404-l.417 that behavioural interventions do not mean replacing other types of actions and putting the responsibility on individuals. 

 Minor Points  

- Expand summaries of key studies to include population and scope. 

  Answer: we now mention sample size and caracteristics of participants in the studies we describe p.8 and 9   

- Correct typographical errors ("informationsession", "unclearer effects"). 

Answer: we did not find the “Informationsession” typos, in our manuscript it appears as two separate words. We did replace “unclearer effects” by “more uncertain effects” p.8 l.144-145. 

- Consider adding an appendix listing key EDCs referenced. 

Answer: we now cite in the paper the list of targeted compounds p.11 l.259-261.  

- Ensure global—not only EU—context is acknowledged. 

Answer: we now broaden the geographical scope of the problem (p.5 l.13-l.16)   

 Conclusion   The manuscript is scientifically sound and makes a valuable contribution. With the revisions outlined above—particularly clarifying the manuscript's purpose, strengthening the causal logic, and refining the language regarding expected outcomes—it will be well-positioned for indexing 

 Answer: we thank the reviewer and hope our modifications matches his expectations

# Reviewer report: Reviewer response for version 1

**DOI:** 10.21956/openreseurope.23544.r66751

**Published:** 2026-02-10

**Authors:** Moses O Alfred, Oyedolapo Bamigboye

**Affiliations:** 1 Redeemer’s University, Ede, Nigeria; 2 Ilishan-Remo, Babcock University School of Basic and Applied Sciences (Ringgold ID: 309474), Ede, Ogun State, Nigeria

Reviewers' comments:

 This manuscript addresses an important and timely challenge: the persistent reliance on information-based approaches to reduce exposure to endocrine-disrupting chemicals (EDCs), despite strong behavioural science evidence that knowledge alone is insufficient to drive sustained behaviour change. By integrating established behavioural models and illustrating their application through the HYPIEND project, the authors make a valuable contribution to intervention design and policy-oriented environmental health research. The manuscript is conceptually sound and well aligned with its stated objectives. However, several aspects of framing, structure, and language would benefit from clarification to ensure that readers clearly understand the paper’s conceptual and methodological nature, its intended contribution, and the boundaries of the claims being made.

 The comments below are intended to strengthen the manuscript without altering its core message.

1. The manuscript currently sits between a narrative review, a conceptual framework paper, and a project description. While this hybrid approach is acceptable, the manuscript does not explicitly state its primary contribution, which may lead to misinterpretation by readers expecting empirical results. I suggest that authors explicitly state in the Introduction that the manuscript is a conceptual and methodological contribution aimed at improving the design of EDC exposure-reduction interventions from the behavioural science perspective.
2. Authors are to clarify that HYPIEND is presented as an illustrative, theory-informed intervention model, not as an evaluated or completed trial.
3. Several sections use language that may be interpreted as implying demonstrated effectiveness (e.g., “able to reduce exposure” or “effective interventions”) despite the absence of outcome data. It is suggested that authors use conditional or forward-looking phrasing (e.g., “is designed to,” “is expected to,” “will be evaluated”) when referring to intervention outcomes except where there is sufficient empirical evidence for the conclusion.
4. Authors are to systematically revise the language to clearly distinguish between evidence-based (behavioural theory, prior intervention research) and the expected outcomes of HYPIEND.
5. Although multiple behavioural models are discussed and applied, the rationale for selecting specific frameworks (e.g., HAPA) over others is sometimes implicit. Briefly justify the selection of key behavioural models (e.g., HAPA, BCT taxonomy).
6. The manuscript presents many components of the HYPIEND intervention, but the causal logic linking psychological determinants to behaviour change and exposure reduction is not always explicit. Add a concise theory-of-change description (textual or tabular) that links psychological determinants, behaviour change techniques, behavioural outcomes and reduced exposure. This will enhance internal coherence and make the intervention logic more accessible to practitioners and policymakers.
7. While the manuscript acknowledges regulatory and environmental determinants of exposure, the emphasis on individual and social behaviour could be misinterpreted as shifting responsibility away from structural actors. Explicitly state that behavioural interventions are complementary to regulatory, industrial, and policy-level actions, not substitutes. In addition, authors should discuss how behavioural approaches can enhance the effectiveness of structural measures (e.g., improved compliance, adoption, and risk communication).
8. Equity considerations are mentioned but not fully developed, despite their relevance to behavioural interventions. The manuscript could be improved with a brief discussion of differential capacity to adopt recommended behaviours across socioeconomic groups and of how theory-driven interventions can be adapted to reduce inequities rather than exacerbate them.
9. “unclearer effect” in line 12, paragraph 1 page 5 could be substituted with an alternative word that would make the statement/point clear for readers.
10. Additional information (probably as an appendix of supporting documents) on some identified EDCs may further enhance the robustness of the manuscript
11. Authors referred to some studies but over-summarized them, for example, for statements like “Results showed that it failed to significantly change behaviour”, the scope of the study (population) and the extent to which the study failed could be included to bring context to the discussion and aid comparison.
12. Edit the typo „informationsession“ in paragraph 3 (last line), page 5
13. The scope (reference) of EDCs in the environment could be extended to all global regions

Where applicable, are recommendations and next steps explained clearly for others to follow? (Please consider whether others in the research community would be able to implement guidelines or recommendations and/or constructively engage in the debate)

Yes

Does the article adequately reference differing views and opinions?

Yes

Are all factual statements correct, and are statements and arguments made adequately supported by citations?

Partly

Is the rationale for the Open Letter provided in sufficient detail? (Please consider whether existing challenges in the field are outlined clearly and whether the purpose of the letter is explained)

Yes

Is the Open Letter written in accessible language? (Please consider whether all subject-specific terms, concepts and abbreviations are explained)

Yes

Reviewer Expertise:

Water Quality Monitoring; Water Treatment; Evidence-based policy engagement; Environmental quality monitoring; CO2 conversion; climate

We confirm that we have read this submission and believe that we have an appropriate level of expertise to confirm that it is of an acceptable scientific standard, however we have significant reservations, as outlined above.

# Response

**Published:** 2026-04-07

**Authors:** Lisa Moussaoui

**Affiliations:** Section de Psychologie, Universite de Geneve Faculte de Psychologie et des Sciences de l'Education, Geneva, Geneva, Switzerland

This manuscript addresses an important and timely challenge: the persistent reliance on information-based approaches to reduce exposure to endocrine-disrupting chemicals (EDCs), despite strong behavioural science evidence that knowledge alone is insufficient to drive sustained behaviour change. By integrating established behavioural models and illustrating their application through the HYPIEND project, the authors make a valuable contribution to intervention design and policy-oriented environmental health research. The manuscript is conceptually sound and well aligned with its stated objectives. However, several aspects of framing, structure, and language would benefit from clarification to ensure that readers clearly understand the paper’s conceptual and methodological nature, its intended contribution, and the boundaries of the claims being made. 

 The comments below are intended to strengthen the manuscript without altering its core message.   

- We thank the reviewers for their precise and constructive recommendations. Below we detail how we address each point. 

  #1 The manuscript currently sits between a narrative review, a conceptual framework paper, and a project description. While this hybrid approach is acceptable, the manuscript does not explicitly state its primary contribution, which may lead to misinterpretation by readers expecting empirical results. I suggest that authors explicitly state in the Introduction that the manuscript is a conceptual and methodological contribution aimed at improving the design of EDC exposure-reduction interventions from the behavioural science perspective. 

 Answer: We agree that the first version of the manuscript was at the crossroads between several types of papers. We now explicitly state that the primary contribution is conceptual and not experimental (p.5-6 l.44- 46) 

 #2 Authors are to clarify that HYPIEND is presented as an illustrative, theory-informed intervention model, not as an evaluated or completed trial. 

 Answer: The HYPIEND project is now more clearly presented as a trial in progress with no experimental data available at this time and presented for an illustrative goal. (p.5 l.41-44) 

 #3 Several sections use language that may be interpreted as implying demonstrated effectiveness (e.g., “able to reduce exposure” or “effective interventions”) despite the absence of outcome data. It is suggested that authors use conditional or forward-looking phrasing (e.g., “is designed to,” “is expected to,” “will be evaluated”) when referring to intervention outcomes except where there is sufficient empirical evidence for the conclusion.

  Answer: We reformulated the sentences that were misleading about the experimental evidence (p.5 l.36; p.13 l.363; p.15 l.420-421, l.425) 

 #4 Authors are to systematically revise the language to clearly distinguish between evidence-based (behavioural theory, prior intervention research) and the expected outcomes of HYPIEND. 

 Answer: We corrected instances of unclear wording (in the abstract, twice “evidence-based”; p.10 l.242-243; l.249; p.13 l.363-364) 

 #5 Although multiple behavioural models are discussed and applied, the rationale for selecting specific frameworks (e.g., HAPA) over others is sometimes implicit. Briefly justify the selection of key behavioural models (e.g., HAPA, BCT taxonomy). 

 Answer:  the use of BCT taxonomy is now justified in the text (p.11 l.274-278), as well as the HAPA framework (p.11 l.262-266 and l.267-270). 

 #6 The manuscript presents many components of the HYPIEND intervention, but the causal logic linking psychological determinants to behaviour change and exposure reduction is not always explicit. Add a concise theory-of-change description (textual or tabular) that links psychological determinants, behaviour change techniques, behavioural outcomes and reduced exposure. This will enhance internal coherence and make the intervention logic more accessible to practitioners and policymakers. 

 Answer: we added in appendix a theory-of-change schema for each study to explicit the causal logic of our project  

 #7 While the manuscript acknowledges regulatory and environmental determinants of exposure, the emphasis on individual and social behaviour could be misinterpreted as shifting responsibility away from structural actors. Explicitly state that behavioural interventions are complementary to regulatory, industrial, and policy-level actions, not substitutes. In addition, authors should discuss how behavioural approaches can enhance the effectiveness of structural measures (e.g., improved compliance, adoption, and risk communication). 

 Answer: we thank the reviewers for this comment and confirm that it would be a misinterpretation of our paper to consider it as putting responsibility on individuals. This paper was written in the context of a policy brief (now cited p.14 l.410) aiming to urge professionals and policy makers to design better communication on EDCs. In order to avoid misinterpretation of our paper, we now mention regulatory and industrial actions p.6 l.83-84 as essential. We also stress in the discussion that the point of the paper is not to put responsibility on individuals and mention other types of policy categories p.14 l.404-412. We discuss how behavioural approach can support or impede other types of measures effectiveness p.14 l.412-417. 

 #8 Equity considerations are mentioned but not fully developed, despite their relevance to behavioural interventions. The manuscript could be improved with a brief discussion of differential capacity to adopt recommended behaviours across socioeconomic groups and of how theory-driven interventions can be adapted to reduce inequities rather than exacerbate them. 

 Answer: We now discuss equity considerations in more depth, p.6 l.72-77 (in terms of inequity in exposition to EDCs), and p.13 l.347-358 (for inequity in access to alternatives).  #9 “unclearer effect” in line 12, paragraph 1 page 5 could be substituted with an alternative word that would make the statement/point clear for readers. 

 Answer: we reworded this sentence for better clarity (p.8 l.144-145) 

 #10 Additional information (probably as an appendix of supporting documents) on some identified EDCs may further enhance the robustness of the manuscript 

 Answer: We now specify the list of compounds that are included in the HYPIEND project p.11 l.259-261 

 #11 Authors referred to some studies but over-summarized them, for example, for statements like “Results showed that it failed to significantly change behaviour”, the scope of the study (population) and the extent to which the study failed could be included to bring context to the discussion and aid comparison.   

 Answer: We now describe more in details the studies cited in the paper p.8 l.155-157, l.162, p.9 l.172, l.175, l.182. More specifically, we mentioned the sample size for each study, described more clearly the results of the PREVED study, and calculated effect sizes for the two studies described as successful for comparative purposes. 

 #12 Edit the typo „informationsession“ in paragraph 3 (last line), page 5 

 Answer: Unfortunately, we did not find the typo in the paper, and wonder if it is a problem of display depending on the software in which the manuscript was open? 

 #13 The scope (reference) of EDCs in the environment could be extended to all global regions 

 Answer: Thank you for this comment. We had to shorten the paper for a previous submission and focused on EU data, but we definitely agree that it is a global issue. We have now extended the geographical scope of the introduction (p.5 l.13-16)

# Reviewer report: Reviewer response for version 1

**DOI:** 10.21956/openreseurope.23544.r69001

**Published:** 2026-02-06

**Authors:** Mukaddes T Miral

**Affiliations:** 1 Istanbul Kültür University, Istanbul, Turkey

The article is a comprehensive study targeting groups at risk for endocrine disruptors, emphasizing that education alone is insufficient for creating behavioral change and discussing the necessity of interventions such as motivational interviewing in addition to education. However, in terms of content, it gives the impression of being somewhere between a review and a protocol study. Following a long introduction and a detailed literature review, two study protocols, HYPIEND and SWPBIS, are introduced; however, the aims, scope, and methods of these protocols are not sufficiently clear.

 It is not explicitly stated whether the main purpose of the study is to present two study protocols or to defend the hypothesis that "education alone is insufficient for behavioral change against endocrine disruptors." Furthermore, it is known that most existing studies in the literature also demonstrate that education alone is insufficient and suggest different interventions. Therefore, the original contribution of this study needs to be more clearly presented.

 The article notes that previous studies are insufficient because they focus primarily on information transfer; however, the proposed HYPIEND and SWPBIS protocols also appear to be largely based on similar approaches. The protocol does not clearly define which interventions (training, motivational interviewing, role modeling, etc.) will be implemented, which measurement tools will be used for evaluation, and what kinds of results are expected after the intervention.

 Accordingly, it is suggested that the introduction and literature review sections be shortened; and that the structure of the protocol, planned interventions, expected outcomes, and evaluation methods be presented in a clearer, more systematic, and simpler language. This would make the purpose, scope, and scientific contribution of the study more understandable.

Where applicable, are recommendations and next steps explained clearly for others to follow? (Please consider whether others in the research community would be able to implement guidelines or recommendations and/or constructively engage in the debate)

Partly

Does the article adequately reference differing views and opinions?

Yes

Are all factual statements correct, and are statements and arguments made adequately supported by citations?

Yes

Is the rationale for the Open Letter provided in sufficient detail? (Please consider whether existing challenges in the field are outlined clearly and whether the purpose of the letter is explained)

Partly

Is the Open Letter written in accessible language? (Please consider whether all subject-specific terms, concepts and abbreviations are explained)

Partly

Reviewer Expertise:

endocrine disruptors, women's health, adolescent health, reproductive health

I confirm that I have read this submission and believe that I have an appropriate level of expertise to confirm that it is of an acceptable scientific standard, however I have significant reservations, as outlined above.

# Response

**Published:** 2026-04-07

**Authors:** Lisa Moussaoui

**Affiliations:** Section de Psychologie, Universite de Geneve Faculte de Psychologie et des Sciences de l'Education, Geneva, Geneva, Switzerland

We thank the reviewer for the valuable comments and explain below how we addressed each of them. 

**Reviewer's comment #1: **The article is a comprehensive study targeting groups at risk for endocrine disruptors, emphasizing that education alone is insufficient for creating behavioral change and discussing the necessity of interventions such as motivational interviewing in addition to education. However, in terms of content, it gives the impression of being somewhere between a review and a protocol study. Following a long introduction and a detailed literature review, two study protocols, HYPIEND and SWPBIS, are introduced; however, the aims, scope, and methods of these protocols are not sufficiently clear. 

 It is not explicitly stated whether the main purpose of the study is to present two study protocols or to defend the hypothesis that "education alone is insufficient for behavioral change against endocrine disruptors." Furthermore, it is known that most existing studies in the literature also demonstrate that education alone is insufficient and suggest different interventions. Therefore, the original contribution of this study needs to be more clearly presented.   

**Answer: **We agree that the original version of the manuscript was unclear in its contribution. Following suggestion from several reviewers, we now more clearly state in the introduction that the paper aims to provide a conceptual discussion of what is needed to trigger behaviour change in order to reduce the exposition to EDCs, and give an illustration of a trial in progress based on theory (p.5-6, l.42-46).  We would like to clarify that both presented studies fall within the HYPIEND project. The SWPBIS is one of the theoretical frameworks used in the study design, along with the HAPA and motivational interviewing.  Finally, we agree that the literature already show that education alone is insufficient, this is exactly the point of the paper: to make readers aware that evidence exist on the need to go beyond mere information provision. We thought that such point needed a publication, not for a researcher audience (because the field is already aware), but for a wider public such as policy-makers and professionals, because it continuously seems that despite this scientific knowledge, many communication campaigns continue to be merely information provision (not only on EDCs but on many other health topics and broader). It might be useful to inform that this paper was published in relation to a policy brief common to the cluster of projects associated with HYPIEND (now cited p.14 l.410).   

**Reviewer's comment #2:** The article notes that previous studies are insufficient because they focus primarily on information transfer; however, the proposed HYPIEND and SWPBIS protocols also appear to be largely based on similar approaches. The protocol does not clearly define which interventions (training, motivational interviewing, role modeling, etc.) will be implemented, which measurement tools will be used for evaluation, and what kinds of results are expected after the intervention. 

 Accordingly, it is suggested that the introduction and literature review sections be shortened; and that the structure of the protocol, planned interventions, expected outcomes, and evaluation methods be presented in a clearer, more systematic, and simpler language. This would make the purpose, scope, and scientific contribution of the study more understandable. **Answer: **We included a theory of change schema to clarify the structure of the intervention (in appendix). Regarding measurement tools, it is not the intention of this paper to provide the details of the study protocol (in terms of outcomes and evaluation methods), since this is going to be published in specific papers with regards to the study protocols. However, we clarified that we expect a positive change in the intervention group, and no change in the control group p.14 l.378-380
